# Liposomal formulation of Galbanic acid improved therapeutic efficacy of pegylated liposomal Doxorubicin in mouse colon carcinoma

**DOI:** 10.1038/s41598-019-45974-7

**Published:** 2019-07-02

**Authors:** Maryam Ebrahimi Nik, Bizhan Malaekeh-Nikouei, Mohamadreza Amin, Mahdi Hatamipour, Manouchehr Teymouri, Hamid Reza Sadeghnia, Mehrdad Iranshahi, Mahmoud Reza Jaafari

**Affiliations:** 1 Nanotechnology Research Center, Pharmaceutical Technology Institute, Mashhad University of Medical Sciences, Mashhad, Iran; 2 Student Research Committee, Mashhad University of Medical Sciences, Mashhad, Iran; 3 Laboratory Experimental Surgical Oncology, Section Surgical Oncology, Department of Surgery, Erasmus Medical Center, Rotterdam, The Netherlands; 4 Natural Products and Medicinal Plants Research Center, North Khorasan University of Medical Sciences, Bojnurd, Iran; 5 Division of Neurocognitive Sciences, Psychiatry and Behavioral Sciences Research Center, Mashhad University of Medical Sciences, Mashhad, Iran; 6 Biotechnology Research Center, Pharmaceutical Technology Institute, Mashhad University of Medical Sciences, Mashhad, Iran; 7 Department of Pharmaceutical Nanotechnology, School of Pharmacy, Mashhad University of Medical Sciences, Mashhad, Iran

**Keywords:** Nanoparticles, Tumour angiogenesis

## Abstract

Galbanic acid (Gba), a *sesquiterpene coumarin*, with strong antiangiogenic activity could serve as an excellent anti-cancer agent. However, Gba is a poor water-solube which hampered its clinical application. In this study, a pegylated liposomal Gba (PLGba) with HSPC/Cholesterol/mPEG_2000_-DSPE (56.2, 38.3, 5.3% molar ratio) was developed by the thin film hydration plus extrusion and calcium acetate gradient remote loading method, to address the issue of poor Gba solubility. Moreover, an integrin-targeting ligand (RGD peptide, cyclo[Arg-Gly-Asp-D-Tyr-Cys]) was post-inserted into liposomes in order to increase Gba cell delivery. Using fluorescently-labeled model liposomes, it was found that the targeting could improve the integrin-mediated cellular uptake of the liposomes *in vitro* in human umbilical vein endothelial cells (HUVECs), and *in vivo* as evidenced by chicken chorioallantoic membrane angiogenesis (CAM) model. It also could enrich the liposome accumulation in C26 tumor. Interestingly, co-treatment with PLGba and pegylated liposomal doxorubicin (PLD, also known as Doxil^®^) had a synergistic and antagonistic antiproliferative effect on the C26 tumor cell line and the normal HUVEC, respectively. In C26 tumor bearing BALB/c mice, the PLGba and PLD combinatorial therapy improved the antitumor efficacy of the treatment as compared to those of single agents. This results have clear implications for cancer therapy.

## Introduction

Cancer chemotherapy has remained a challenge as the present chemotherapeutic agents are accompanied by some serious side effects^1^. Extensive investigations have been conducted to restrict the drug delivery to the tumor environment and minimize the off-target effect^2,3^. Also, the quest for new cancer-treating agents is underway to find a potential agent from nature with differential cytotoxicity between cancer cells and normal cells^4^. To this end, enormous candidates of natural products, including Galbanic acid (Gba), are on the desk to be examined for their medical properties^5–7^.

Gba is a lipophilic *sesquiterpene coumarin* isolated from the roots of various *Ferula* species (Apiaceae)^8^, with multiple useful biological properties, including anti-cancer^9,10^ and antiangiogenic activities^10^. However, the clinical application of Gba is limited due to poor aqueous solubility, fast elimination, and limited bioavailability^8^, which necessitates conducting formulation researches to present a stable colloidal formulation of Gba.

To enhance the solubility of a hydrophobic substance such as Gba, lipid-based drug delivery systems^11,12^, especially liposomes, are among the best candidates; liposomes have already been shown to enhance the circulation time, reduce the systemic toxicity and improve the therapeutic efficacy of a drug. When a stable colloidal liposomal formulation of a drug is injected intravenously (i.v.), it leads to a high concentration of the drug with prolonged drug exposure to tumor tissues and also the minimization of the systemic drug delivery^13–15^. This effect arises out of the large gaps exclusively found between endothelial cells in tumor vessels as well as the lack of lymphatic drainage system in tumor environment that allows the extravasation and entrapment of large nanoparticles, including liposomes. Thanks to this effect, which is putatively known as the “Enhanced Permeability and Retention” (EPR) effect^16,17^, particles with diameter of 200 nm to 1.2 µm depending on tumor type could extravasate from the tumor vasculature in animal models; however, an upper limit of about 200 nm is expressed for successful drug delivery^18–20^. Indeed, various liposomal formulations, including the first approved pegylated liposomal doxorubicin (PLD, commercially known as Doxil^®^), are shown to improve the therapeutic efficacy of drugs with the help of EPR effect^21,22^.

Additionally, it has been shown that targeting liposomes with specific ligands enhance the liposome delivery, and the drug thereof, to the cancer cells overexpressing the corresponding receptors. More specifically, targeting liposome with RGD peptide, which interacts selectively with αvβ3 integrin overexpressed on tumor endothelial cells^23–25^, results in preferential transfer of a drug to cancer cells and improved tumor therapy^26,27^.

To the best of our knowledge, no parenteral dosage form of Gba has been yet investigated for tumor therapy because of its poor water solubility and fast elimination from body fluids. This is the first report on developing a stable colloidal liposomal formulation of Gba, which overcame the solubility problem of this substance and increased the circulation time.

We successfully developed a pegylated liposomal formulation for Gba (PLGba) delivery to the tumor microenvironment. The designed liposomes were modified with an RGD peptide (cyclo [Arg-Gly-Asp-D-Tyr-Cys]). Various features of the liposome were investigated, including liposomal size distribution and physical stability, Gba release, cell association, tissue distribution, and pharmacokinetic profile. Furthermore, the liposomal cell internalization and the associated antiangiogenic activity were investigated in a human umbilical vein endothelial cells (HUVECs) and a chicken chorioallantoic membrane. The antitumor activity of RGD targeted liposomal Gba (RGD- PLGba) as well as PLGba, alone and in combination with PLD, was also examined in C26 colon carcinoma bearing BALB/c mice. We observed that PLGba could improve the antitumor activity of PLD. In conclucion, the PLGba and PLD combinatorial therapy could be a potential effective treatment for colon carcinoma.

## Results and Discussion

### RGD-to-lipid conjugation

**Figure 1:**
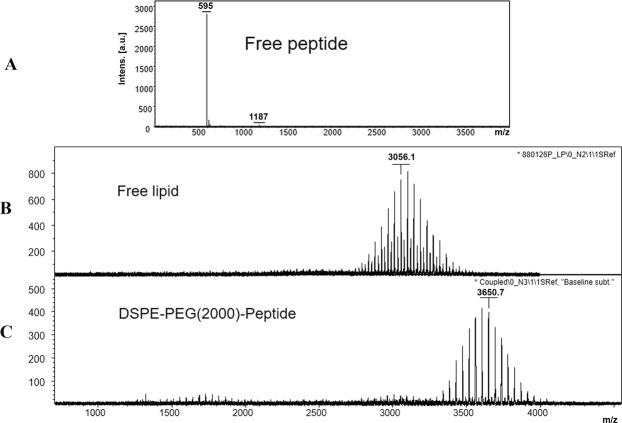
MALDI-TOF mass spectroscopy analysis data for the RGD peptide (**A**), Mal-PEG_2000_-DSPE (**B**), and RGD-lipid (**C**) revealed the consumption of free DSPE-PEG2000-Mal in reaction and formation of the DSPE-PEG-RGD lipopeptide.

The prerequisite step toward the preparation of the RGD-targeted liposomes was to link the RGD peptide covalently to the maleimide-PEG_2000_-DSPE. For this purpose, it was of high importance to determine the efficiency of the conjugation. The efficiency of the coupling was monitored during the reaction period on the TLC, in which the covalent attachment of the lipid to the peptide prevents the lipid spot from moving with the developing solvent of chloroform/methanol/water (90/10/2) on the paper (data not shown). Moreover, given the molecular mass of RGD (595 in Fig. 1A), the MALD-TOF mass spectroscopy data of the lipid-RGD shifted completely to the right from the average molecular weight of 3056.1 (Fig. 1B) to 3650.7 (Fig. 1C) indicating that all the lipids are consumed and linked. This showed that the RGD-lipid that is necessary for the preparation of the liposomes was synthesized.

### Physicochemical stability characterization of the liposomes

**Figure 2:**
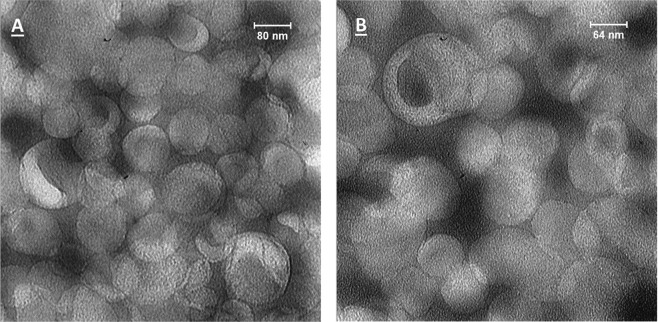
Transmission electron microscopy (TEM) images of negatively stained PLGba at magnification of ×25000 (**A**) and ×31500 (**B**).

The physicochemical stability of the liposomes did not noticeably change during the storage at the time of the liposome preparation. The liposome size distribution and surface charge (z-potential values) remained constant among the preparations (Table 1), indicating that neither Gba addition nor RGD-targeting disturbs the size stability of the liposomes probably due to their same liposomes’ surface charge. The liposome size stability remained unaffected with increasing Gba-to-total lipid ratio and excess Gba was not entrapped in the liposomes and it was colonized on the inner-surface of the glass vials (data are not shown). The liposomes, i.e., empty liposome, PLGba, and RGD-targeted PLGba, had a mean particle size of about 100 nm with narrow particle size distribution (polydispersity index or PDI of <0.150), suitable for the objective of the current study, which is the exclusive particle accumulation in tumor environment based on the EPR effect. The small particle size, morphology, and to some degree the narrow particle size distribution were confirmed as depicted by the TEM graph of PLGba (Fig. 2).

**Table 1:** Physicochemical properties of the liposomes.

Formulation	Nomenclature	Z-average (nm)	PDI^c^	Z- potential (mV)	Gba (mg/ml)	EE^d^%
Gba-liposomes	PLGba	102 ± 12^b^	0.143 ± 0.043	−6.1 ± 4.3	1.8	90 ± 9.3
	RGD-PLGba^a^	104 ± 19	0.095 ± 0.066	−6.5 ± 4.4	1.62	81 ± 8
Fluorescently-labeled model liposomes	Model liposome	106 ± 22	0.031 ± 0.022	−7.0 ± 2.8	—	—
	RGD-model liposome^a^	103 ± 16	0.102 ± 0.035	−7.3 ± 5.4	—	—

^a^RGD-liposomes contained 0.13% molar ratio of the RGD, equivalent to 100 peptide/liposome.

^b^Data are shown as mean ± standard deviation of three independent liposomal preprarations.

^c^Stan ds for polydispersity index.

^d^Stands for encapsulation efficiency calculated according to Equation 1.

The physicochemical stability of the PLGba had also maintained during long-term storage (5 months) at 4 °C (Table 2). Only a marginal increase in mean particle size was observed for the PLGba and other parameters, i.e., PDI, liposome surface charge and the Gba content of the liposomes, remained unchanged. These findings indicate that the PLGba is a liposome formulation with long-term storage physicochemical stability.

**Table 2:** Physicochemical stability of PLGba within storage period in refrigerature.

Time (month)	Z-average (nm)	PDI^b^	Z-potential (mV)	Gba content (mg/ml)
0	102 ± 4^a^	0.078 ± 0.043	−8.0 ± 3.5	1.62 ± 0.23
0.5	103 ± 6	0.086 ± 0.026	−6.2 ± 2.8	1.58 ± 0.18
1	105 ± 8	0.117 ± 0.019	−9.9 ± 2.7	1.47 ± 0.21
2	110 ± 12	0.143 ± 0.025	−7.8 ± 3.1	1.78 ± 0.32
3	111 ± 14	0.121 ± 0.029	−4.3 ± 1.4	1.76 ± 0.36
4	113 ± 23	0.119 ± 0.032	−7.5 ± 1.1	1.80 ± 0.17
5	118 ± 18	0.092 ± 0.042	−5.4 ± 1.9	1.52 ± 0.18

^a^Data are shown as mean ± standard deviation of three independent liposome preprarations.

^b^Stands for polydispersity index.

**Figure 3:**
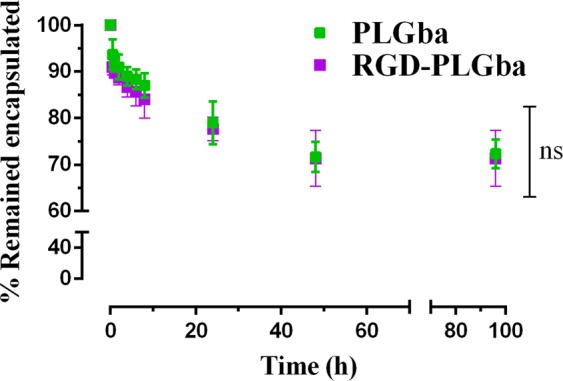
Liposomal Gba content of PLGba and RGD-PLGba incubated at 37 °C in the PBS/FCS buffer for 96 hours. Data are presented as mean ± standard deviation (n = 3). Ns indicates non-significant (p > 0.5).

The incubation of the liposomes in the PBS/FCS medium at 37 °C undermined, to some degree, the physicochemical stability of the liposomes, as it caused the Gba content of the liposomes to be reduced to 70% of their initial content (Fig. 3). This could be attributed to the inter-particular transition of Gba among the liposomes and serum proteins and probably their sedimentation, not to the Gba release to the aqueous medium, considering the calculated octanol-to-water partition coefficient of Gba molecule. The calculated n-octanol-to-water partition coefficient of Gba, expressed as XlogP3-AA, was reported at 5.1, indicating that Gba is about 1 × 10^5^ more hydrophobic than hydrophilic. In another word, the probability of Gba present in an organic phase is about 100,000 times higher than that in an aqueous solution^7^. Given the fact, one could surmise that it is extremely rare for Gba to leave the liposomal membranes and leak to the external aqueous solution. As a result, more than 70% of Gba remained encapsulated in the liposomes, even at 96 h post-incubation in a simulated physiologic medium like the PBS/FCS. Furthermore, it could be inferred that the stealth pegylated liposomes are quite stable to patrol the cargo (Gba) in systemic circulation to reach the tumor.

### Liposome- cell association study

The next step was to evaluate the liposome-cell association in an endothelial cell line with high integrin expression, similar to those present in most tumor vasculature. To this end, HUVEC was taken for the study. Due to the technical complication of the Gba measurement, RGD-targeted, and non-targeted model liposomes were used, in which the hydrophobic fluorescent tracking dye of lissamine rhodamine B sulfonyl PE (Liss-Rhod PE) was used instead of Gba. In fact, integration of the trace amount of dye such as Liss-Rhod PE within the lipid bilayer of liposomes, has resulted in a very robust and stable assembly, causing no alteration of liposome properties, such as their interaction with cells or cellular-uptake^28^.

**Figure 4:**
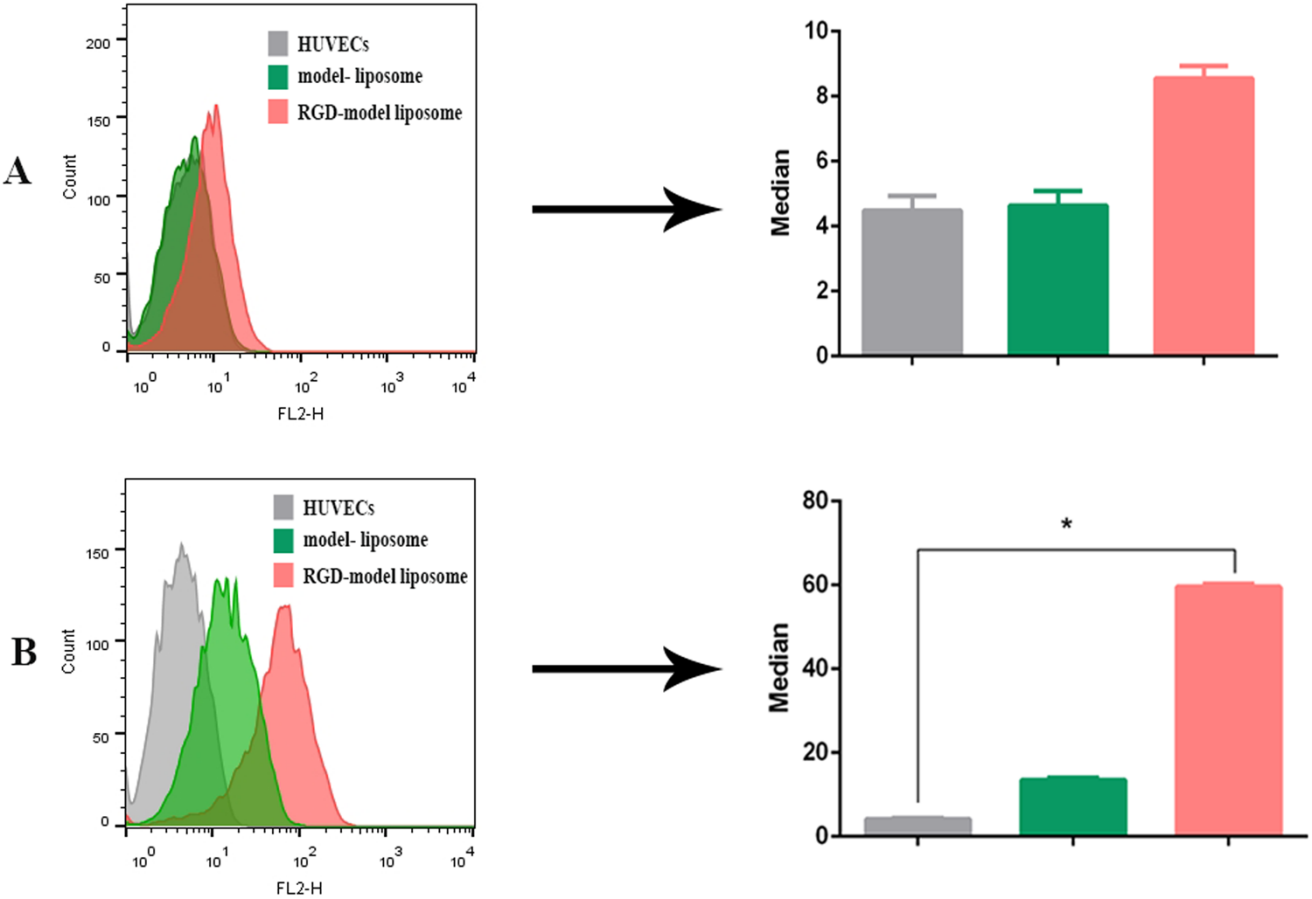
*In vitro* cellular binding affinity (**A**) and liposome-cell association (**B**) of the RGD-targeted and non-targeted fluorescently-labeled model liposomes in HUVECs at 4 °C and 37 °C, respectively. Data are shown as mean ± standard deviation of three independent experiments. * indicates significant differences (p < 0.05).

As shown in Fig. 4, liposome-cell interaction was demonstrated by cellular fluorescence after 37 °C incubation and model liposomes retention were also observed when no significant cellular fluorescence was detected at 4 °C. Also, all the gates for cyclometer of HUVECs as well as scatter plot can be found as Supplementary Fig. S4. The fluorescence intensity measument was represented that median was increased 1.8-fold at 4 °C (Fig. 4A) and it rose by 4.4-fold at 37 °C (Fig. 4B) in samples treated with the RGD-targeted model liposome as compared to the samples treated with the non-targeted liposomes. It could be inferred from these findings that targeting PLGba with the RGD peptide could significantly enhance Gba delivery to the integrin-overexpressing endothelial cells present in tumor’s vasculature^29^ as we have proved that RGD peptide could internalize into the HUVECs and tumor vasculature through integrin mediated endocytosis by confocal microscopy and intravital microscopy in our previous study^30,31^.

How much Gba introduction in the form of PLGba formulation could exert an antiproliferative effect on endothelial and C26 tumor cells is dealt in the following.

### PLGba antiproliferative property

It was found that f-Gba was markedly less toxic than f-Dox (Table 3). Both PLGba and f-Gba (provided in DMSO) shared similar cytotoxicity, indicating that Gba encapsulation into the liposome and Gba emulsion in DMSO are equally available to C26 cells. Concerning Dox, PLD exhibited significantly lower toxicity than f-Dox, as evidenced in our previous study. PLGba was about 40-times less toxic than PLD, considering the IC_50_ values in HUVECs. While f-Gba, PLGba, and RGD-PLGba showed virtually identical toxicities against C26 cell, they differed in toxicity against HUVEC. The antiproliferative activity of Gba increased in HUVECs in the form PLGba and it was further increased upon targeting PLGba with the RGD peptide. Such cell-specific enhanced toxicity could be attributed to the targeting event, in which targeting PLGba with RGD enhanced the delivery of the fluorescently-labeled liposomes to HUVEC, as evidenced in Fig. 4B.

**Table 3:** Half-maximal inhibitory concentration (IC_50_) of the drugs in free and liposomal form following single drug-treatment and cotreatment of the cells with PLD and PLGba.

Treatment	HUVEC (µM)	C26 (µM)
f-Gba	207 ± 5.7^a^	84.9 ± 4.8
f-Dox	0.09 ± 0.05^b^	0.043 ± 0.02^b^
PLD	3.1 ± 0.07^b^	1.9 ± 0.03^b^
PLGba	124.4 ± 3.8^b^	82.3 ± 5.3
RGD-PLGba	48.3 ± 2.2^b,c^	79.12 ± 6.6
PLD + PLGba	0.9 ± 0.2/ 137.7 ± 23.5^d^	0.7 ± 0.04/56.3 ± 3.4
Combination index	1.1 ± 0.2	0.7 ± 0.04

^a^Data are shown as the mean ± standard deviation (n = 8).

^b^Indicates a significant difference compared to f-Gba.

^c^Indicates a significant difference compared to PLGba.

^d^The first and second set of figures are the mean ± standard deviation of the IC_50_ values for PLD and PLGba, respectively.

Co-treatment of the cells with PLGba and PLD resulted in different interactive antiproliferative outcomes in the cells (Table 3). According to Table 3, the co-treatment reduced the required dose of Gba and Dox to restrict the cell growth by 50% in the cancer C26 cells, considering that the IC_50_ values reduced from 82.3 to 56.3 µM for PLGba and from 1.9 to 0.7 µM for PLD. Moreover, the CI was achieved <1. These denote a synergistic antiproliferative effect on the cancer cells. On the other hand, the co-treatment led to a decreased cytotoxicity in the normal HUVEC cell line, in which the PLGba and PLD showed an antagonistic relationship (CI > 1).

### Antiangiogenic activity

**Figure 5:**
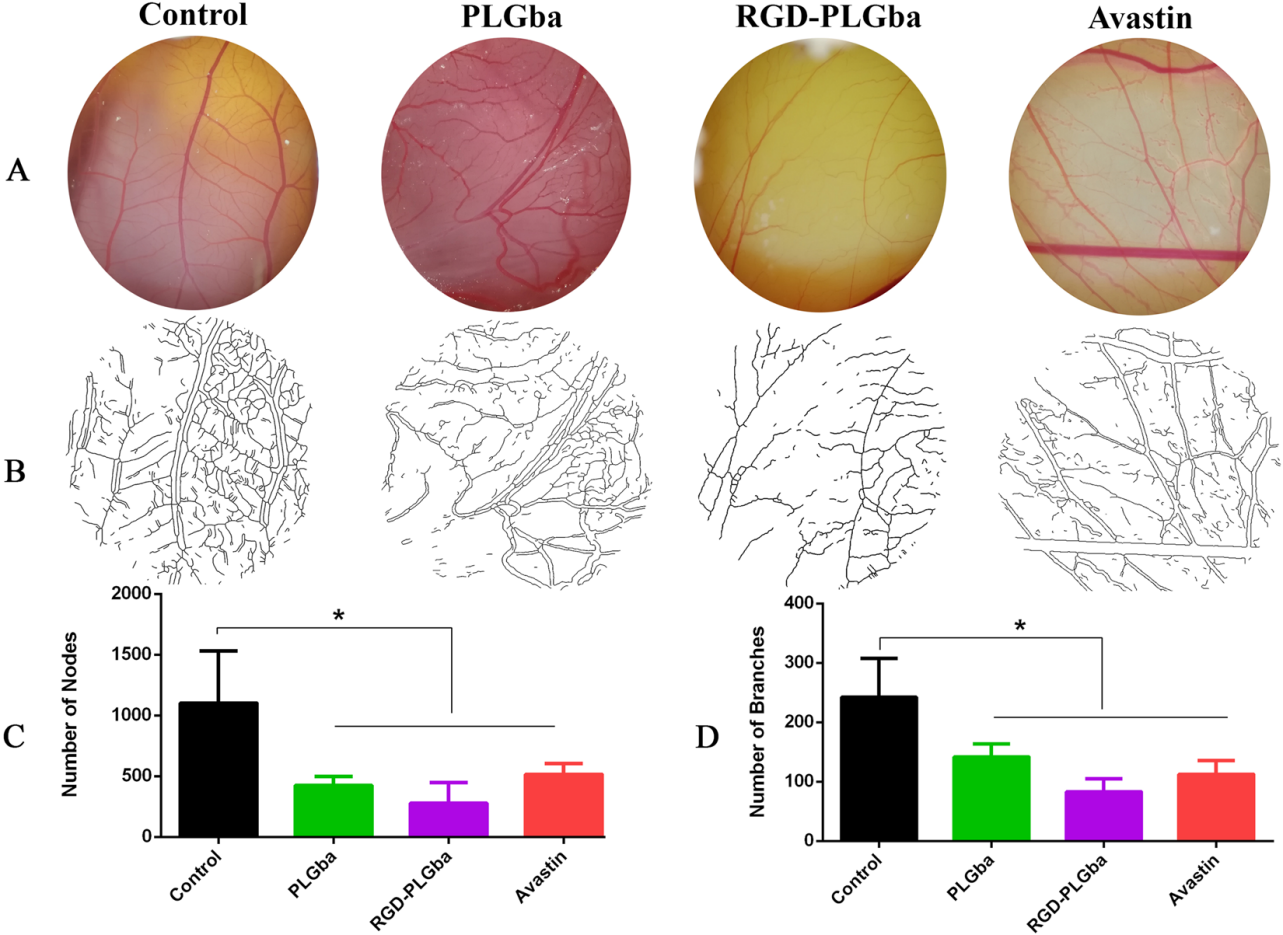
CAM vasculature development on day 12 of egg development treated with PLGba, RGD-PLGba, and Avastin (**A**) shows the stereomicroscope of the CAM vessels, (**B**) is the quantitative analysis of the chicken CAM using NIH ImageJ with the angiogenesis analyzer plugin. (**C**) and (**D**) are the numbers of vascular nodes and branches. Data are expressed as mean ± standard deviation (n = 6). * indicates significant difference as compared to control (p < 0.05).

Antiangiogenic activity is one of the biological features of Gba mentioned in the literature, which was also determined in our study for the RGD-PLGba and PLGba. Indeed, the antiangiogenic activity of Avastin^®^ as a gold standard positive control was demonstrated. The antiangiogenic activity of f-Gba was not determined due to the poor water solubility and bioavailability of Gba to the CAM. Moreover, although Gba were dissolved in organic solvents like ethanol and DMSO, these solvents were found to be highly toxic to CAM; therefore, we were unable to measure f-Gba antiangiogenic properties on CAM. The injection of PLGba, RGD-PLGba, and Avastin^®^ significantly limited normal neovascularization in CAM at 20 µM dose injection (Fig. 5). The injection of the liposomes at higher doses severely blocked the development of new vessels in CAM completely.

Figure 5A shows the stereomicroscopic photograph of CAM vessels on day 12 of egg development, 72 h after treatment. In the control group, normal neovascularization with clear regular directional patterns was observed in CAM, whereas in PLGba, RGD-PLGba, and Avastin^®^ eggs, the CAM vessels became less dense with an irregular pattern or even blind-ended.

Quantitative analysis of the vasculature was evaluated in the chicken CAM with NIH Image J with the angiogenesis analyzer plugin (Fig. 5B). The number of nodes and branches of the vasculature significantly reduced in CAM vessels of the PLGba, RGD-PLGba, and Avastin^®^ treatment groups as compared to the non-treated control group (Fig. 5C,D).

The CAM is formed by the fusion of the chorion and allantois membrane on day 4^th^ of incubation, which plays an important role in gas exchange with the extraembryonic environment. It has a great number of vascular network that forms a continues surface in direct contact with the egg shell^32^. The entire CAM was readily accessible for local administration of the liposomes on day 8^th^ of the incubation. It is stated that there is rapid angiogenesis in CAM from 8^th^ to 11^th^ day^33^, a period which was suitable to test our agents.

In addition to the CAM model assay, histological analysis of the tumor tissues from *in vivo* chemotherapy study could povide more comprehensiv insight on Gba antiangiogenic mechanism. However, the CAM model has several advantages over histological analysis. These include: (1) The complications due to presence of artifacts in prefixation, fixation, tissue processing, staining steps in histological analyses are avoided by direct visualization of the entire CAM; (2) CAM assay are more easily reproducible and can be undertaken with much higher throughput; (3) As the CAM assay is a closed system, the half-life of many experimental molecules tends to be much longer in comparison to animal models, allowing experimental study of potential anti-metastatic compounds that are only available in small quantities which are important determinants for choice of a method^33–35^; (4) Since chick embryo’s immune system is immature in the early phase of development; so non-specific inflammatory reactions and consequent angiogenesis would not occur^33^. This is while in mouse model, the immune system is mature^35^ and thus not suitable for studying anti-angiogenic substances; (5) CAM model assay provides a general overview of vascular networks over a large area which grants a deeper insight into the tissues without any artifacts. This is while, histological analyses provide microscopic images of small sections of the tumor that may contain artifacts.

Because of all of the above mentioned reasons, we believe that using CAM assay provide a more accurate and comprehensive view of the Gba’s antiangiogenic activity in a large are of the chicken chorioallantoic membrane. In other hands, the CAM model assay could be used as a good alternative methods of study on determination of the antiangiogenic mechanism of Gba.

Taken these into account, the limitation in the vasculature development of CAM could be attributed to the antiangiogenic properties of the Gba-liposomes (PLGba and RGD-PLGba).

### Animal studies

#### Pharmacokinetic and biodistribution of liposome

In this study, the fluorescently-labeled model liposomes was used for studying pharmacokinetic and biodistribution of liposomes in place of PLGba and directly detection of Gba concentration by the mentioned spectroscopy, HPLC or MS in the tissues or plasma. As the main goal of this study is design and development of an effiecient delivey system for Gba inorder to target the tumor vasculatures by RGD-peptide, it is crutial to track Gba cariers (RGD-targeted and non-targeted model liposomes) in the body instead of tracking the delivery of the Gba. In other words, it would be impossible to distinguish between free Gba and the liposomal form of this substance in plasma and/or tissues by studying the biodistribution of PLGba and analyzing the Gba concentration directly by the above mentioned methods whereas, studying biodistribution of the model liposomes may give the opportunity of compare RGD-targeted and non-targeted model liposomes in the body more accurately. As we demonstrated that RGD-targeted and non-targeted liposomes had similar leakage stability, it was expected for both of these carriers to have a same payload in the body.

Although studying the biodistribution of PLGba instead of the model liposome would provide useful insight, there is a big challenges on the way of analyzing the concentration of Gba using HPLC. Gba is detectable at wavelength of 328 nm which is within the UV range.This is while, several organelles in the cells of the host also emit signals in this wavelength range, causing a lot of background signal in the final result. This fact makes result interpretation very challenging.

Indeed, in this study a simple and fast screening method was developed using Lissamin-Rhodamin PE fluorescent dye for labeling the RGD-targeted and non-targeted liposomes, to: (1) study the biodistribution of the Gba carrier and (2) explore whether the targeting ligand shows satisfactory performance. This pilot investigation sets the grounds for a more comprehensive study on the *in vivo* behavior and mechanisms of Gba and its therapeutic effects. Such comprehensive study would incorporate MS analysis for Gba biodistribution determination.

Hence, it was more preferable to use fluorescently labeled lipids into the liposomal membrane as it is well-established and has been used in several studies^36–39^ to determine the drug carrier behavior within the host.

**Figure 6:**
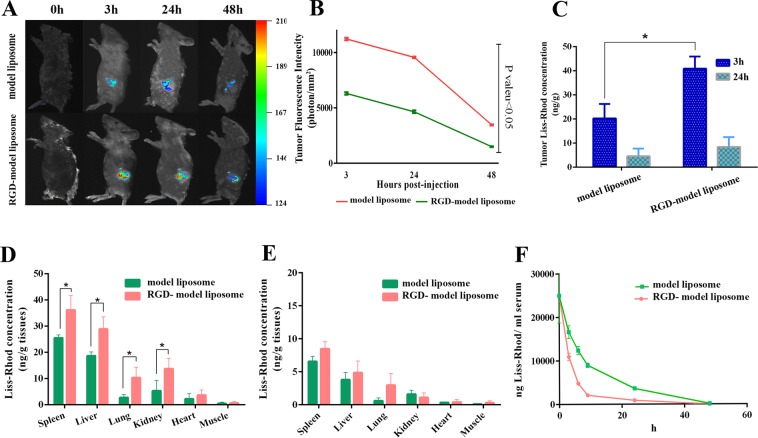
*In vivo* fluorescence image and the related fluorescent intensity of tumor, tissues, and serum in BALB/c mice bearing C26 colon tumor injected with RGD-targeted and non-targeted fluorescently-labeled model liposomes. (**A**) and (**B**) are the real-time fluorescence images and the associated fluorescence intensity data at the mentioned time points. (**C**) indicates the concentration of the fluorescent dye (ng/g tissues) in tumor at 3 and 24 h post-injection. (**D**) and (**E**) are the tissues concentrations of the dye at 3 and 24 h post-injection, respectively; and (**F**) is the serum concentration of the dye within 48 h post-injection. Data are shown as mean ± standard deviation (n = 3). * indicates significant differences (p < 0.05).

The RGD targeted and non-targeted model liposomes exhibited significantly different real-time tumor accumulation (Fig. 6A,B). RGD-model liposomes accumulated further in tumor and the fluorescence signal was 1.8, 2.1, and 2.3 folds higher than that of the non-targeted at 3, 24, and 48 h post-injection, respectively. Overall, upon targeting the liposomes with the RGD peptide, the level of fluorescent signal enhanced in tumor site, especially 3 h post-injection (Fig. 6C).

Perhaps due to dissociation of Liss-Rhod fluorescent dye from the liposome, a decline in fluorescence intensity at 24 h compared to 3 h was observed. In a recent study by Munter *et al*. 2018, they claim that 50% dissociation is corresponded to all fluorescently labeled lipids in the outer leaflet of the liposomal membrane^40^. Also, lipid exchange could be another factor which caused a lower accumulation of model liposomes at 24 h post-injection. Because of these variables which existed in different time points, it was more accurate to compare model liposomes with RGD-model liposomes at each time point instead of comparing these two groups in different time points. That said, Fig. 6C purposely shows the comparison between accumulation of model liposomes versus accumulation of RGD-model liposomes in tumor at 3 h post-injection. The analysis of spleen and liver data revealed a faster clearance for RGD-model liposome at 3 h post-injection (Fig. 6D). The concentration of the fluorescent dye in the spleen and liver for the RGD-targeted liposomes was 1.41- and 1.54-folds greater than non-targeted liposomes, respectively (p < 0.05). Interestingly, the concentration of the fluorescent dye in the tissues of the tumor (Fig. 6C), spleen, and liver (Fig. 6D) at 3 h were all above 20 ng/g, while the only visible signal was observed from the tumor. This was possibly due to the fact that the tumor was inoculated subcutaneously on the right flank of the mice, which is near the surface of the body whereas, spleen and liver were buried in the peritoneum, a spot too deep to give out any signal even in ventral position.

Moreover, a higher concentration of the dye was found in the kidney and lung tissues of mice treated with RGD-targeted liposomes (Fig. 6D). No significant difference was found in other tissues at 3 h post-injection and in all tissues at 24 h post-injection (Fig. 6D, E). Furthermore, tissue distribution of model liposomes did not conduct at 48 h post-injection due to the fact that result of *in vivo* real-time fluorescent images of mice showed a huge reduction of fluorescent intensity at this time point.

Although targeting the liposome with the RGD enhanced the tumor accumulation of the liposome, it also enhanced the accumulation of the liposome in some other tissues, which could be attributed to the expression of integrin in these tissues. In this regard, the markedly higher accumulation of the targeted liposomes in the tumor could be attributed to the integrin overexpression in C26 tumors, which could attach to the RGD peptides on the surface of the liposomes. Moreover, it is reported that spleen, liver, lung, and kidney contains a high level of integrin receptors in their vasculature, which might explain the higher accumulation of the targeted liposomes^41,42^. The fluorescent signal significantly decreased in the serum of mice treated with RGD-model liposome as compared to that of the non-targeted (Fig. 6F). Table 4 shows the parameters relating to the fluorescent signal decay for such reduction in serum (Equation 4). There was a strong correlation between the empirical data and the predicted data considering the parameters relating to the goodness of fitness, i.e., the coefficient of determination (R^2^ > 0.9), the high absolute sum of squares (Absolute SS), and the low standard deviation of the residuals (Sy.x). Both liposomes had the same C_max_ values, confirming that the mice received an equal dose of the model liposomes. The fluorescent signal decay rate (K) was significantly higher in the serum of mice treated with RGD-model liposome than that of the non-targeted (0.274 versus 0.121, Table 4). Furthermore, the half-life of the fluorescent signal and the corresponding area under the curve (AUC) decreased significantly in the serum of mice treated with RGD-model liposome compared to that of the non-targeted from 287 to 87 µg*h/ml. The decreased level of the fluorescent signal in serum could be attributed to the higher accumulation of the RGD-model liposomes in tissues as compared to those of the non-targeted. In other word, targeting liposomes with the RGD peptide could lead to their enhanced blood clearance rate and the cargo thereof. Accordingly, similar distribution and kinetic profiles are imagined for the RGD-PLGba and PLGba.

**Table 4:** Serum pharmacokinetic data relating to the coefficients of the Equation 4 and the goodness of fitness.

Best-fitted values	Model liposome	RGD-model liposome
Y_0_ or Cmax (µg/ml)	24.5	25
Plateau (µg/ml)	1.08	0.01
K	0.121	0.274
Half-life (h)	5.7	2.5
AUC^a^ (µg*h/ml)	287	87
**Goodness of fit**		
d.f.^b^	15	15
R^2^	0.92	0.94
Absolute SS	94.6	79.4
Sy.x.	2.5	2.3

^a^Area under the curve.

^b^Degrees of freedom.

#### Chemotherapy study

**Figure 7:**
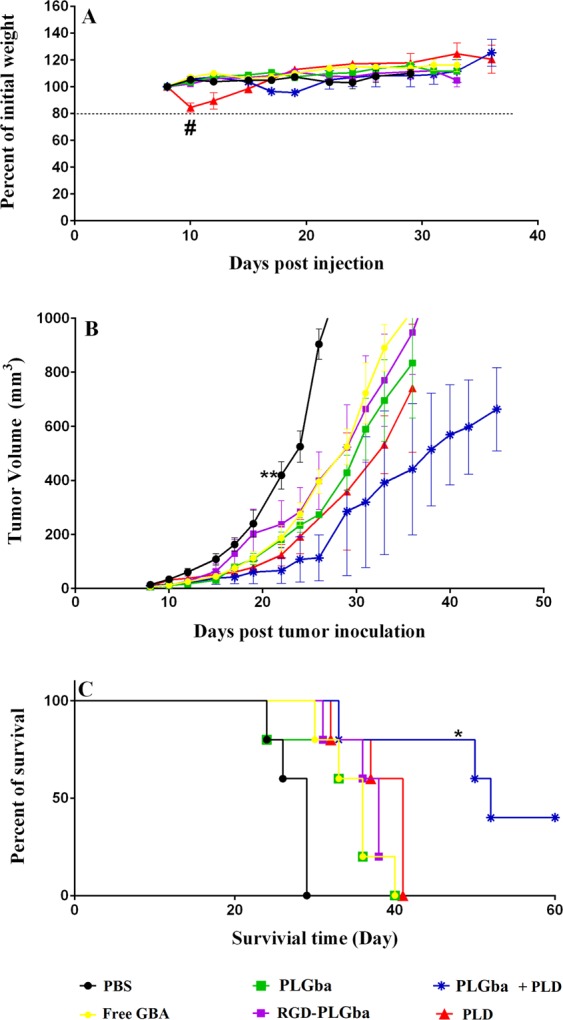
*In vivo* therapeutic efficacy of f-Gba, Gba-liposomes, PLD and PLD + PLGba combination in C26 tumor-bearing mice treated with multiple doses of Gba (10 mg Gba/kg) and a single dose of PLD (10 mg Dox/kg). The Gba-liposomes did not change the body weight (**A**), but they delayed tumor growth up to day 22^th^ post-injection (**B**) and increased the lifespan, especially in mice treated with PLD + PLGba (**C**). Data are expressed as mean ± standard deviation (n = 5). ^#^Indicates a significant difference between PLD and control group, ** indicates a significant difference between all treatment groups and the control group, and *indicates a significant difference between PLD + PLGba treatment group and all treatment groups (p < 0.05).

All Gba dosage forms, including f-Gba, led to a significant therapeutic outcome (Fig. 7). While they did not result in any weight loss in mice (Fig. 7A), they controlled tumor growth to some extent (Fig. 7B) and increased the lifespan of mice (Fig. 7C) as compared to those of control group that received dextrose 5% solution. The group received a single dose of PLD displayed a significant weight loss compared to the control group, whereas the combination therapy with PLD and PLGba resulted in no body weight loss. This indicates that Gba might provide some useful protective benefits, which help the animals to tolerate the Dox-related side-effects.

Combination treatment with PLD and the PLGba caused significant tumor growth delay as compared to the control group (Fig. 7B). In this regard, the combination therapy showed slightly improved antitumor activity as compared to the drugs alone. The improved antitumor efficacy following the combination therapy could arise from the cytotoxic property of PLD and antiangiogenic activity of Gba as demonstrated in the cytotoxicity and CAM assays, respectively. This is in agreement with the report of Ma, J. *et al*. who found that a chemotherapy combination with an antiangiogenic agent leads to improved efficacy of cancer therapy^43^. Furthermore, additional histological analyses of the tumor tissues could provide more comprehensive insight on Gba antiangiogenic mechanism. On the other hand, this study sets the grounds for more comprehensive study on the *in vivo* behavior and mechanisms of Gba and its therapeutic effects.

Treatment with PLD significantly increased the lifespan of mice as compared to the control group (Fig. 7C). Although treatment with f-Gba, PLGba, and even RGD-PLGba only marginally led to increased lifespan, the PLD-chemotherapy combination with PLGba enhanced the survival time of mice significantly, even as compared to the PLD monotherapy. For instance, PLD injection was shown to increase the median survival time from 29 days to 41 days, and the combination therapy enhanced it further to 52 days (Table 5). The combination therapy improved TTE and enhance TGD by 71%, while neither the free-form nor the liposomal form of Gba changed the survival time and the related parameters.

**Table 5:** Therapeutic indices for C26- tumor-bearing mice injected with the liposomes and drugs.

Treatment in colon carcinoma	Median survival time	Time to endpoint	Tumor growth delay (%)	Increased lifespan (%)
Control	29	27.2 ± 1.2^a^	—	—
f-Gba	36	34.7 ± 2.1	34	24
PLGba	36	32.7 ± 6.1	26	24
RGD-PLGba	38	35 ± 4	35	31
PLGba + PLD	52	44.5 ± 11.3^b,c^	71^c^	79^c^
PLD	41	36.6 ± 4	34.32	41.4

^a^Data are shown as the mean ± standard deviation (n = 5).

^b^Indicates a significant difference compared to control.

^c^Indicates a significant difference compared to PLGba.

According to the biodistribution data analysis, it was found that Gba was accumulated in the liver, spleen and tumor tissues, the same organs in which Dox is reportedly accumulated when it is injected to the blood circulation in the form of PLD. Since Gba had very low cytotoxicity against C26 cells, low levels of Gba in the tumor could not exert a cytotoxic effect on these cells as studied by the fluorescently-labeled model liposomes; therefore, it is far-fetched for Gba to reach an effective cytotoxic concentration in tumor environment using the current liposome formulation and technology. This fact was confirmed by the survival analysis as it was found that the Gba-liposomes were ineffective in inhibiting tumor size growth. However, Gba could also be accumulated in the vital organs like liver and spleen in the form of the Gba-liposomes. It is reported that Gba could display some cytoprotective effect against some cellular stresses, especially on hepatocellular tissue^5^. The accumulation of the Gba-liposomes could provide some cytoprotective effect against the cell stresses caused by PLD accumulated in these organs. This might also explain why the combination therapy did not reduce the animals’ body weight as the PLD monotherapy did. Therefore, liposomal Gba could be applied in the management of chemotherapy-related side-effects, which merits investigation.

## Conclusion

In the present study, we developed a method to encapsulate efficiently poorly water-soluble agent (Gba) into a stable pegylated liposomal formulation and modified the liposomes with an RGD peptide to improve Gba delivery to the tumor. The physicochemical properties of the liposomes, including particle size, particle charge, and drug content were optimized so that they could remain constant during the long-term storage period in refrigerator and incubation in the PBS/FCS at 37 °C. Although the fluorescently-labeled RGD-targeted liposome (as the model of RGD-PLGba) represented an improved tumor-oriented liposome delivery in C26 colon tumor model, RGD-PLGba could only inhibit the tumor growth slightly (not statistically significant compared to the mice treated with PLGba). This phenomenon could be due to the suboptimal accumulation of Gba in tumor tissue, as the non-tumoral tissues attracted part of the injected RGD-PLGba by having integrin receptors on their blood vessels endothelial cells. Hence, the objective of tumor-oriented drug delivery with RGD peptide is accomplished at the cost of the enhanced drug delivery to the normal tissues. To explore the combinatorial effect of PLGba on the toxicity reduction of chemotherapeutic liposome formulation of PLD, multiple doses of PLGba was used in combined with a low dose of PLD. It was observed that PLGba could improve the outcome of the chemotherapy even with a reduced dose of PLD (as low as 10 mg/kg). The survival rate in tumor bearing mice was increased when the mice were treated with combinatorial therapy. Also, mice treated with both PLGba and PLD lost less weight compared to the mice treated with only PLD. Taken together, addition of PLGba to the PLD treatment regimen reduced the side effects of this chemotherapeutic agent and showd a more robust tumor growth inhibition effect. Whether or not PLGba could improve the efficiency of cancer chemotherapy with other chemotherapeutic agents and control the hepatotoxicity of the current liposomal chemotherapeutics are intriguing to be answered, which merit investigation.

## Materials and Methods

### Materials

Gba was a gift from Dr. Mehrdad Iranshahi, Department of Pharmacognosy and Biotechnology, Faculty of Pharmacy, Mashhad University of Medical Sciences, Mashhad, Iran. The Gba was previously extracted from the herbal root of *Ferula szowitsiana* and its chemical structure was characterized^44^. Fertilized white leghorn chicken eggs were purchased from Symorgh Company (Mashhad, Iran).

Hydrogenated soy phosphatidylcholine (HSPC) and methoxy polyethylene glycol (Mw 2000)-distearyl phosphatidylethanolamine (mPEG_2000_-DSPE) were purchased from Lipoid (Ludwigshafen, Germany). Maleimide-PEG_2000_ distearyl phosphatidylethanolamine (Mal-PEG_2000_-DSPE) and the fluorescent tracking dye 1,2-dioleoyl-sn-glycero-3-phosphoethanolamine-N-(lissamine rhodamine B sulfonyl) ammonium salt were obtained from Avanti polar lipids (USA). Cholesterol was supplied from Sigma-Aldrich (St Louis, MO). RGD peptide (cyclo [Arg-Gly-Asp-D-Tyr-Cys]) at 99.9% purity was from Peptides International Inc (Louisville, USA). Isopropanol was purchased from Merck (Darmstadt, Germany). The commercially available pegylated liposomal doxorubicin (Doxil^®^) was supplied from Behestan Darou Company (Tehran, Iran). The commercially available Bevacizumab (Avastin^®^, Stivant 400 mg/16 ml) was purchased from AryoGen Pharmed company (Tehran, Iran). All other solvents and reagents were of a chemical grade.

With respect to cell culture media and reagents used with cells, RPMI 1640 culture medium was purchased from Sigma-Aldrich (St. Louis, MO), diphenyltetrazolium bromide (MTT) was from Promega (Madison, WI), and endothelial cell growth supplement (ECGS) and collagenase type I were from Sigma–Aldrich (St. Louis, MO, USA). Fetal calf serum (FCS), penicillin-streptomycin, L-glutamine and HEPES buffer were obtained from GIBCO (UK).

### Conjugation of the RGD to maleimide-PEG2000-DSPE

The RGD peptide was attached covalently to maleimide-PEG_2000_-DSPE in an anhydrous medium^45^. For this purpose, the peptide (10 mg/ml dissolved in DMSO) and lipid (10 mg/ml dissolved in chloroform) was mixed at 1.2/1 peptide/lipid molar and incubated overnight at room temperature in a dark under an argon atmosphere and continuous agitation. The linkage efficacy was monitored by silica thin layer chromatography (TLC) with a developing solvent of chloroform/methanol/water (90/10/2) and iodine vapor exposure. Then, the solvents were removed under a warmed nitrogen stream and the tube’s content was freeze-dried overnight^46^. The resulting white powder was re-suspended in an appropriate amount of deionized water. Subsequently, the probable uncoupled peptide-cysteine residues were blocked by adding an excessive amount of cysteine to the reaction tube. The tube’s content was dialyzed against NaCl (1 M) in a dialysis cassette (Pierce, Rockford, IL) with 30 kDa molecular weight cut-off (MWCO) followed by excessive dialysis against distilled water. The conjugation efficacy was confirmed by MALDI-TOF (Ultraflex III MS, Bruker Daltonics).

### Liposome preparation

The liposomes were prepared by thin film hydration and an extrusion method. The nomenclature and physicochemical properties of the liposomes are given in Table 1. All liposomes have the same lipid composition as that of PLD, which is HSPC, Chol, and mPEG_2000_-DSPE at 56.2, 38.3, and 5.3% molar ratio, respectively. The lipids were dissolved in ethanol at 65 °C and diluted 10-times gently with a calcium acetate solution (250 mM, pH 7.3) to reach a final total lipid concentration of 100 mM under continuous vortex at 65 °C. Subsequently, the mixture was passed through polycarbonate membranes of 0.4, 0.2, 0.1, and 0.05 μm pore size (Avestin, Canada). The prepared liposome formulation was then dialyzed against HEPES-buffered sucrose (HBS; 10 mM HEPES, 300 mM sucrose, pH 6.0) using a 12–14 kDa MWCO dialysis cassette. Finally, the liposome was incubated with Gba (2 mg Gba/ml liposome) at 65 °C for 1 hour. The excess Gba was removed by centrifugation (14000 × g, 5 min) followed by dialysis (×2) against HBS.

For fluorescently-labeled model liposome, the fluorescent tracking dye was added to the liposome formulation at 0.2% molar ratio of total lipid, along with the other lipids in a flask, from which the organic solvent was removed by the rotary evaporator and freeze-dryer. Then the lipid film was hydrated with HBS and finally, the resultant mixture passed through the polycarbonate filters as explained for PLGba.

Both PLGba and the model liposome were modified with the RGD peptide through the post-insertion method, in which a peptide-lipid micelle is merged into a liposome membrane at a gel-to-liquid crystalline phase transition temperature (Tm) of the liposomal membrane to achieve the targeted-liposome^45^. For this, an appropriate amount of the RGD-lipid micelle was added to the liposomes’ tube and incubated at 60 °C for four h with an occasional shake.

### Liposome characterization

The mean hydrodynamic diameter, Poly-Dispersity Index (PDI) and zeta potential of liposomes wase measured by a Dynamic Light Scattering instrument (Nano-ZS; Malvern, UK). Moreover, the morphology and size of the liposomes were determined by a Leo 912 AB transmission electron microscope (Zeiss, Jena, Germany) opeating at voltage of 120 kV.

The total phospholipid content was determined according to Bartlett phosphate method^47^. Encapsulation Efficiency (EE %) was calculated measuring the Gba concentration before removal of excess Gba from PLGba with the centrifugation and dialysis and after that as per the following equation:

1 $$EE\, \% =(\frac{Gba\,concentration\,post-removal\,of\,excess\,Gba}{Initial\,Gba\,concentration})\times 100$$

Gba concentration of PLGba was measured at 328 nm with a UV-visible spectrophotometer (SPEKOL 1300; Analytik Jena, Germany) after dissolving 0.1 ml of the liposome in 0.9 ml methanol.

### The shelf-life and *in vitro* physicochemical stability of PLGba

The long-term physicochemical stability of PLGba was determined for five-month storage period in a refrigerator (4 °C) and in phosphate-buffered saline/FCS (PBS/FCS; 50/50, v/v) for four days at 37 °C, respectively. The shelf-life stability was monitored by the dynamic light scattering instrument every month. To examine the physicochemical stability, PLGba was subjected to dialysis in a 30 kDa MWCO dialysis cassette against 100 ml PBS/FCS. At several time intervals, samples were drawn from the dialysis bag and centrifuged at 14000 × g for 10 min, from which first 0.1 ml of the supernatant was dissolved in 0.9 ml methanol and then, Gba concentration was measured with spectrophotometry at 328 nm.

### Cell culture

C26 colon carcinoma (Eppelheim, Germany) was grown in RPMI 1640 culture medium supplemented with 10% FCS, 100 IU/ml penicillin, and 100 mg/ml streptomycin. The primary HUVECs were cultured in Human Endothelial-SFM (Gibco, UK) supplemented with 20% newborn calf serum, 10% human serum, 20 ng/ml basic fibroblast growth factor (bFGF), and 100 ng/ml epidermal growth factor (EGF). HUVECs used for the experiment were between 3^rd^ and 6^th^ passages. All cell lines were incubated in a humidified incubator at 37 °C containing 5% CO_2_. The cells were detached by trypsin-EDTA solution (Gibco, UK). The cell viability was evaluated via trypan blue dye exclusion before each experiment^48^.

### Liposome- cell association study

The liposome-cell association was examined via flow cytometry (BD FACS Calibur^TM^, BD Biosciences, San Jose, USA) on the FL2 channel with the detector in logarithmic mode (FL2-H). For this purpose, HUVECs were seeded in a flat-bottomed 24-well plate at 10^6^ cells/well. Following overnight incubation, the cells were treated with the fluorescently-labeled model liposomes at 37 °C and 4 °C for three h at the liposome’s total lipid concentration of 100 nmole phospholipid/ml. The cells were washed ×3 with ice-cold PBS, detached with 0.1 ml of the Trypsin-EDTA solution, transferred to 2 ml tubes, and centrifuged at 800 × g for 5 min. Then, the cell pellets were washed ×3 with PBS containing 0.1% FCS, and re-suspended. Finally, the fluorescent intensity was measured with the flow cytometry.

### Cell toxicity

The antiproliferative effect of the RGD-targeted and non-targeted PLGba, Gba suspension in dimethyl sulfoxide (DMSO, 0.03%; shown as f-Gba), PLD, and Dox solution in saline (shown as f-Dox) was measured in the normal HUVEC and cancer C26 cell lines using MTT assay. Moreover, the antiproliferative effect of the combination of PLGba and PLD was measured at 40 Gba/1 Dox mole drug ratio. For this, HUVEC and C26 cell were seeded at 5000 cells/well in 96 flat-bottomed well plates. Following overnight incubation at 37 °C, the corresponding cell culture media were replaced with a fresh FCS-free medium containing ½ serial dilutions of the agents and incubated further for 72 h in the incubator. Similarly, for the combination therapy, ½ serial dilutions of PLGba and PLD was added to the well at the fixed mole ratio of 40 Gba/1 Dox. Subsequently, 20 µl of MTT solution (5 mg/ml in PBS) was added to the wells and incubated for 3 hours in the incubator. Then, the medium was removed and the cells were dissolved completely in 0.2 ml DMSO. Finally, the absorbance of the wells was recorded at 550 nm using a Multiskan plus plate reader (BioTek EL 800; BioTek Instruments, Bad Friedrichshall, Germany).

The relative cell growth inhibition (R) was calculated as follows:

2 $$R=1-(\frac{{A}_{test}-{A}_{blank}}{{A}_{control}-{A}_{blank}})$$

Where A_test_ and A_control_ were the absorbance values of the cells treated with the test reagents and the culture medium (negative control), respectively. A_blank_ was the absorbance value of the MTT solution added in the cell-free wells (positive control). IC_50_ was then calculated using CalcuSyn version 2 software (BIOSOFT, Cambridge, UK).

Moreover, the interactive response, i.e., synergistic, antagonistic and additive response, of the combination therapy was determined using the following combination index (CI) equation (Equation 3):

3 $$CI=\frac{{D}_{Gba/Gba+Dox}}{{D}_{Gba}}+\,\frac{{D}_{Dox/Gba+Dox}}{{D}_{Dox}}$$

Where D_Gba_/_Gba +Dox_ shows the dose of Gba at a specific inhibitory response, e.g., IC_50_, IC_75_, IC_90_, etc, when Gba is used in the combination with Dox, while D_Gba_ is the dose of Gba at that response when Gba is used alone. Similarly, D_Dox_/_Gba +Dox_ shows the dose of Dox at a given inhibitory response when Dox is used in the combination with Gba, while D_Dox_ is the dose of Dox at that inhibitory response when Dox is used alone. CI < 1, CI = 1, and CI > 1, shows respectively a synergistic, additive, and antagonistic effect.

### Antiangiogenic activity

The antiangiogenic activity of the RGD-targeted, non-targeted PLGba, and Avastin^®^ as a gold standard positive control was assessed using a chick chorioallantoic membrane (CAM) assay. For this purpose, the eggs were first kept in a Multiquip Incubator (Model E2, US) at 37 °C with 60% humidity for eight days. Then, under aseptic condition, a small square window was made in the eggshell (1 × 1 cm), the incised part was removed, 20 µl of the liposomes (20 µM) and Avastin^®^ (20 µM) was injected into the CAM, and the window was capped by the removed shell and fixed with an adhesive tape. For negative control, only PBS was injected into the CAM. The eggs were incubated in the incubator for further four days. Finally, on day 12 of the chicken embryo development, the vasculature of the CAM was photographed with a stereo microscope (LABOMED luxeo 4Z zoom, USA), and the pictures were analyzed by NIH Image J with the Angiogenesis plugin.

### Animal Study

The animal experiment consisted of the study of the chemotherapy-related outcomes and the examination of the serum and tissue distribution of the fluorescent dye in the form of the model liposomes. This study was approved by the Institutional Ethical Committee and Research Advisory Committee of Mashhad University of Medical Sciences, under the protocol number of 930933 and all animal experiments and methods were carried out in accordance with the relevant guidelines and regulations approved by the ethical committee. Furthermore, according to the Guide for the Care and Use of Laboratory Animals, mice were euthanized when they met the euthanasia criteria, including dramatic body weight loss (>20% of initial weight), tumor volume of >1000 mm^3^, or inability to feed.

### Pharmacokinetic and biodistribution study

Tumor model was C26 colon carcinoma in female BALB/c mice (4–6 weeks old). The mice were injected subcutaneously in the right flank with 3 × 10^5^ C26 cells/mouse. When tumors reached 100–200 mm^3^ in volume, the mice received i.v. single dose of the fluorescently-labeled model liposomes at 25 µg dye/mouse. The liposome-associated fluorescent signal accumulation in the tumor was monitored in tranquilized (100 mg ketamine/kg; 10 mg xylazine/kg) mice using the imaging technology of KODAK *In Vivo* Imaging System F Pro (Eastman Kodak Company; molecular imaging system; USA, Excitation: 560 nm, Emission: 583 nm). The mice were imaged both at ventral and lateral positions to acquire signals from the tumor as well as major organs (i.e., spleen and liver). Then images were analyzed by KODAK Molecular Imaging Software which is limited to report the results using “photon counts per mm^2^” unit as this unit has been also repoted in a recent study by Jang C. *et al*.^49^. However, the fact that the same software settings (e.g. KVP, exposure time, FOV, F-stop, etc.) havev been used for all of the experiments, the reported values can be used for comparing the different cases. That said, for quantifying the amount of dye in tumor and major tissues, two groups of mice were sacrificed at 3 and 24 h post-injection, and different tissues were removed and weighted, including heart, lung, tumor, one of the kidneys, a part of the liver and spleen (nearly 150 mg). They were then placed into 2 ml polypropylene microvials (Biospec, OK) containing 1 ml isopropanol/HCl (90/10 v/v, 0.075 M) and zirconia beads and homogenized by a Mini-Bead beater-1 (Biospec, OK). Finally, the fluorescent concentration was measured with spectrofluorimetry against the dye standard curve prepared in tissues of a mouse, previously injected with dextrose 5% solution^50^.

Similarly, the blood samples were taken from mice via retro-orbital bleeding at 3, 6, 12, 24, and 48 h post-injection. The sera were diluted in the acidified isopropanol and stored overnight at 4 °C. The dye concentration in serum was measured spectrofluorimetry against the standard curve. The pharmacokinetic analysis was conducted by fitting the fluorsecent dye concentration time-course data to a non-linear one-phase decay exponential equation (Equation 4) shown below:

4 $$Y={({Y}_{0}-Plateau)}^{-KX}+Plateau$$

where Y_0_ and Plateau are the Y values at zero and infinite times, respectively and K is the rate constant, expressed in reciprocal of the X-axis time unit (h−1).

### Chemotherapy study

The chemotherapy study included f-Gba, RGD targeted and non-targeted PLGba, PLD and PLGba + PLD therapy, in which the body weight change, tumor size growth, and the incident cancer- and chemotherapy-related death were followed. For this, 4–6 week old female BALB/c mice were injected subcutaneously (SC) in the right flank with 3 × 10^5^ C26 cells/mouse. Throughout the following days, the mice were inspected for tumor emergence, and the mice with palpable tumor (approximately 10 mm^3^) were randomly divided into different groups (four per group).

The f-Gba, RGD-targeted, and non-targeted PLGba groups received the agents twice a week for three consecutive weeks at 10 mg Gba/kg via vein tail. The PLD group received a single dose of PLD intravenously (i.v.) at 10 mg Dox/kg body weight on the very day the tumors appeared as well as multiple injections of dextrose 5% solution (0.2 ml/mouse) during three consecutive weeks. Mice in the combination group received the single dose injection of PLD and the multiple dose injections of the PLGba via vain tail. And finally, the control group received the multiple injections of the dextrose 5% solution for three consecutive weeks.

Mice were followed at regular day intervals regarding body weight change and tumor volume; calculated from three orthogonal diameters of the tumor as follows: (a × b × c) × 0.5. Mice were euthanized when they met the euthanasia criteria, including dramatic body weight loss (>20% of initial weight), tumor volume of >1000 mm^3^, or inability to feed^51^. The experiment was continued until the number of subjects in the last group was lower than the group’s median. Time to end point (TTE) for each mouse, that is, the time (expressed in day) taken for the tumor to reach 1000 mm^3^ in volume, was calculated by fitting the tumor volume time-course data to a log-linear equation and it was used to construct survival graphs. Also, tumor growth delay (expressed in percentage term) was calculated for each group as follows:

5 $$TGD \% =(\frac{T-C}{C})\times 100$$

where T and C are the mean TTE for the treatment and control groups, respectively.

### Statistical analysis

Statistical data analysis was conducted using GraphPad Prism 6 software (San Diego, CA, USA). The log-ranked test was used to find the differences among groups using TTE values between the groups. The two-tailed statistical analysis was performed at a significance level of 0.05. The pharmacokinetic data analysis was performed by fitting the empirical data to a one-phase decay exponential equation (equation 4), using the least square fitting method. The same method was used to calculate the IC_50_ value for the tested agents with CalcuSyn version 2 software (Biosoft, UK). One-way ANOVA and Newman-keuls multiple comparisons were used for other comparisons. For tumor growth experiment, when a tumor volume passed 1000 mm^3^, the final tumor volume recorded for the animal mean size was repeatedly used at the subsequent time points, according to.

## Supplementary information

Supplementary figure
