# Author Correction: Liposomal formulation of Galbanic acid improved therapeutic efficacy of pegylated liposomal Doxorubicin in mouse colon carcinoma

**DOI:** 10.1038/s41598-020-60918-2

**Published:** 2020-02-27

**Authors:** Maryam Ebrahimi Nik, Bizhan Malaekeh-Nikouei, Mohamadreza Amin, Mahdi Hatamipour, Manouchehr Teymouri, Hamid Reza Sadeghnia, Mehrdad Iranshahi, Mahmoud Reza Jaafari

**Affiliations:** 1 Nanotechnology Research Center, Pharmaceutical Technology Institute, Mashhad University of Medical Sciences, Mashhad, Iran; 2 Student Research Committee, Mashhad University of Medical Sciences, Mashhad, Iran; 3 Laboratory Experimental Surgical Oncology, Section Surgical Oncology, Department of Surgery, Erasmus Medical Center, Rotterdam, The Netherlands; 4 Natural Products and Medicinal Plants Research Center, North Khorasan University of Medical Sciences, Bojnurd, Iran; 5 Division of Neurocognitive Sciences, Psychiatry and Behavioral Sciences Research Center, Mashhad University of Medical Sciences, Mashhad, Iran; 6 Biotechnology Research Center, Pharmaceutical Technology Institute, Mashhad University of Medical Sciences, Mashhad, Iran; 7 Department of Pharmaceutical Nanotechnology, School of Pharmacy, Mashhad University of Medical Sciences, Mashhad, Iran

Correction to: *Scientific Reports* 10.1038/s41598-019-45974-7, published online 02 July 2019

This Article contains errors. The colours in Figure 6B are inverted; the correct Figure 6 appears below as Figure 1.

**Figure 1:**
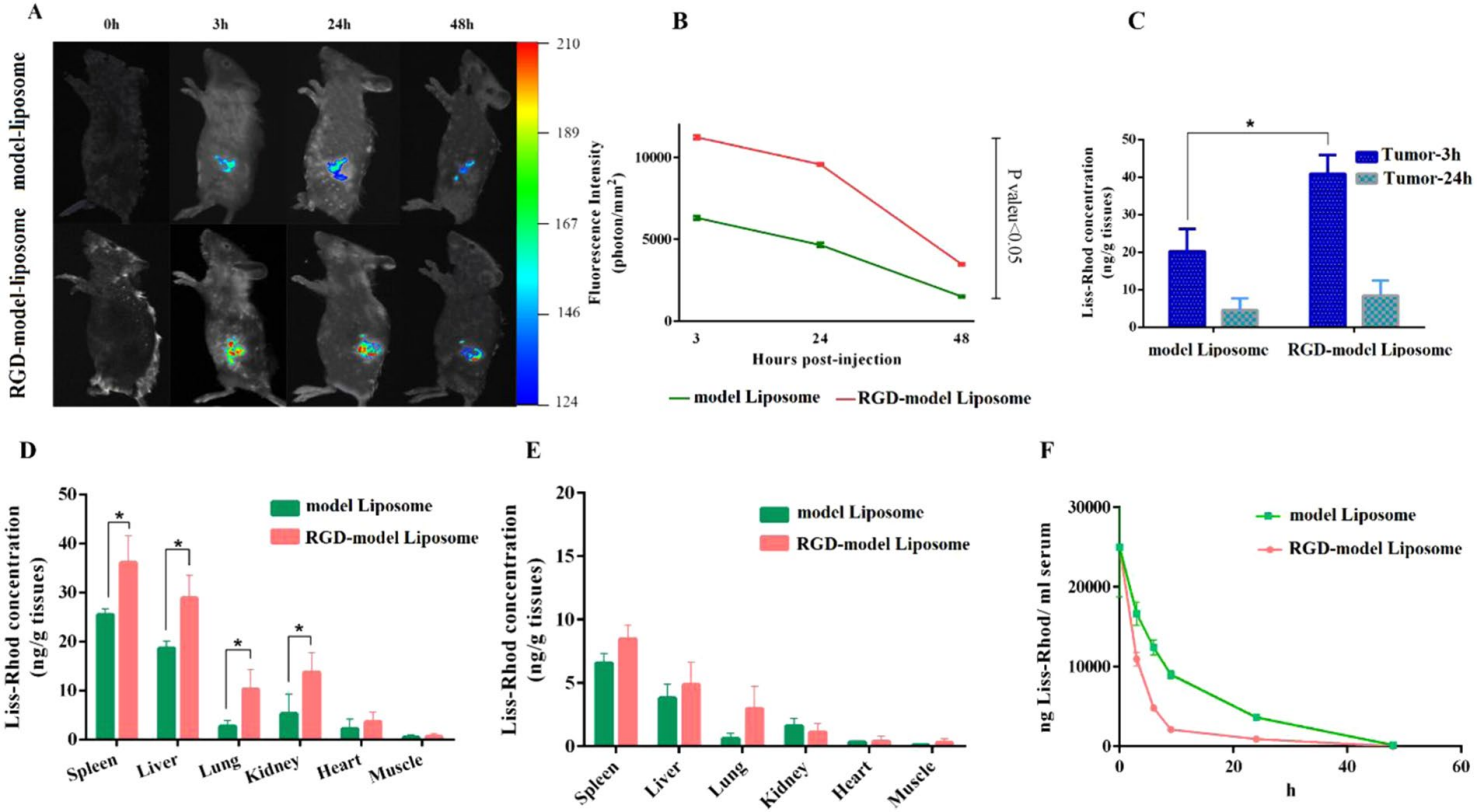
.

In addition, the total phospholipid content is not provided. The text in the Results and Discussion subsection ‘Physicochemical stability characterization of the liposomes’,

“The liposomes, i.e., empty liposome, PLGba, and RGD-targeted PLGba, had a mean particle size of about 100 nm with narrow particle size distribution (polydispersity index or PDI of <0.150), suitable for the objective of the current study, which is the exclusive particle accumulation in tumor environment based on the EPR effect. The small particle size, morphology, and to some degree the narrow particle size distribution were confirmed as depicted by the TEM graph of PLGba.”

should read:

“The liposomes, i.e., empty liposome, PLGba, and RGD-targeted PLGba, had a mean particle size of about 100 nm with narrow particle size distribution (polydispersity index or PDI of <0.150), suitable for the objective of the current study, which is the exclusive particle accumulation in tumor environment based on the EPR effect. Moreover, the total phospholipid concentration of the liposomes was 56.6 ±1.8 mM which agreed with the expected phospholipid concentration (60 mM). The small particle size, morphology, and to some degree the narrow particle size distribution were confirmed as depicted by the TEM graph of PLGba.”

Finally, information on extruder type and extruding times is incomplete. The text in the Materials and Methods subsection ‘Liposome Preparation’,

“Subsequently, the mixture was passed through polycarbonate membranes of 0.4, 0.2, 0.1, and 0.05 μm pore size (Avestin, Canada).”

should read:

“Subsequently, the mixture was extruded (Mini Extruder, Lippex extruder, USA/Canada) by passing 11 times through polycarbonate membranes of 0.4, 0.2, 0.1, and 0.05 μm pore size (Avestin, Canada).”

