# Supplementary material for: Liposomal formulation of Galbanic acid improved therapeutic efficacy of pegylated liposomal Doxorubicin in mouse colon carcinoma
Source: Sci Rep. 2019 Jul 2;9:9527. doi: 10.1038/s41598-019-45974-7 (PMC6606580; doi:10.1038/s41598-019-45974-7)
Supplement: Supplementary file 1 — Supplementary figure [file 41598_2019_45974_MOESM1_ESM.pdf]

Liposomal formulation of Galbanic acid improved therapeutic efficacy of pegylated liposomal Doxorubicin in mouse colon carcinoma

Maryam Ebrahimi Nik[1,2], Bizhan Malaekeh-Nikouei[1], Mohamadreza Amin[3], Mahdi Hatamipour[1], Manouchehr Teymouri[4], Hamid Reza Sadeghnia[5], Mehrdad Iranshahi[6], Mahmoud Reza Jaafari[6,7*]

[1] Nanotechnology Research Center, Pharmaceutical Technology Institute, Mashhad University of Medical Sciences, Mashhad, Iran.

[2] Student Research Committee, Mashhad University of Medical Sciences, Mashhad, Iran.

[3] Laboratory Experimental Surgical Oncology, Section Surgical Oncology, Department of Surgery, Erasmus Medical Center, Rotterdam, The Netherlands.

[4] Natural Products and Medicinal Plants Research Center, North Khorasan University of Medical Sciences, Bojnurd, Iran

[5] Division og Neurocognitive Sciences, Psychiatry and Behavioral Sciences Research Center, Mashhad University of Medical Sciences, Mashhad, Iran.

[6] Biotechnology Research Center, Pharmaceutical Technology Institute, Mashhad University of Medical Sciences, Mashhad, Iran.

[7] Department of Pharmaceutical Nanotechnology, School of Pharmacy, Mashhad University of Medical Sciences, Mashhad, Iran

*Corresponding author: Dr. Mahmoud Reza Jaafari, Biotechnology Research Center, Nanotechnology Research Center, School of Pharmacy, Mashhad University of Medical Sciences, Mashhad, Iran. E-mail: Jafarimr@mums.ac.ir
Tel: ++98513 8823255; Fax: +98 513 8823251.

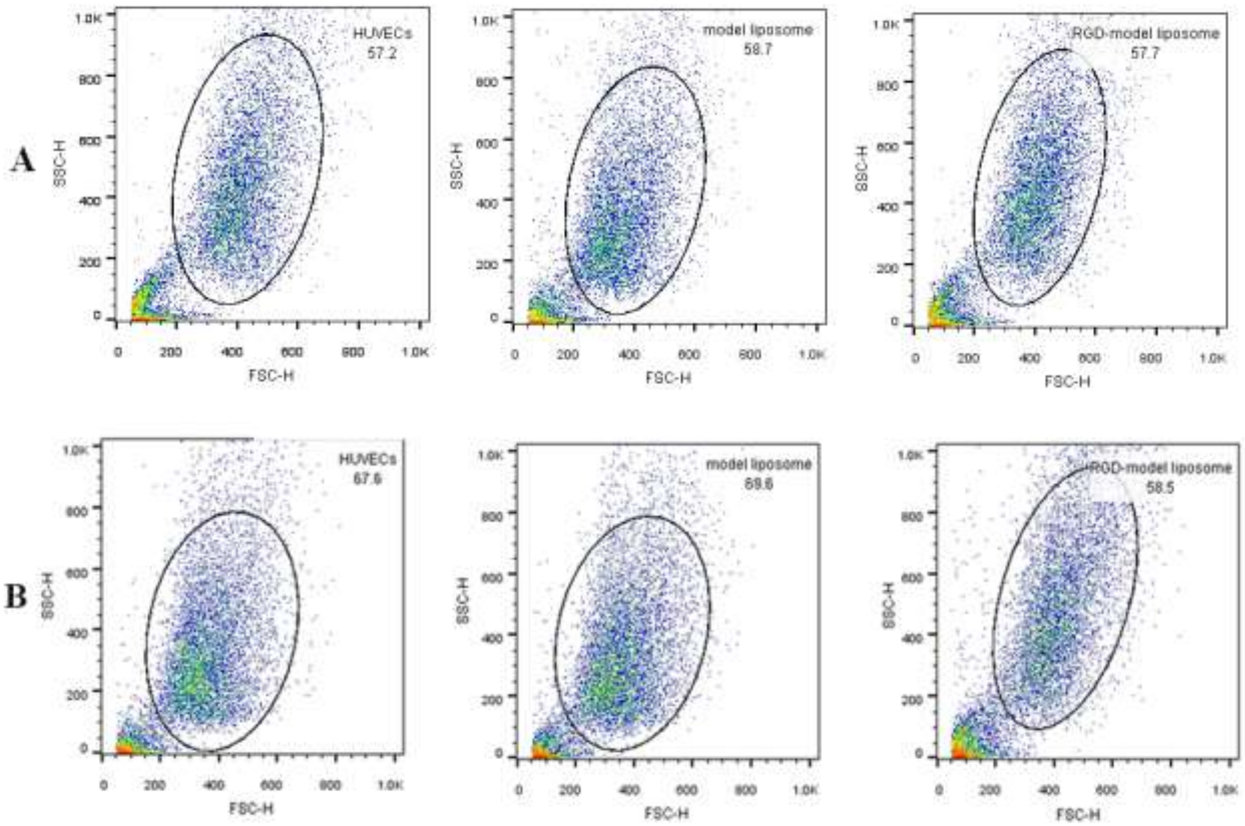

Supplementary Figure S4: Flow cytometry of HUVECs treated with unstained buffer, model liposomes, and RGD-model liposomes after incubation at 4°C (A) and 37°C (B). Each spot represents a single live cell.
